# Concomitant sinus histiocytosis with massive lymphadenopathy (Rosai-Dorfman Disease) and diffuse large B-cell lymphoma: a case report

**DOI:** 10.1186/1752-1947-2-70

**Published:** 2008-03-05

**Authors:** James C Moore, Xiaohui Zhao, Edward L Nelson

**Affiliations:** 1 Department of Medicine, Division of Hematology/Oncology, University of California at Irvine, School of Medicine, Irvine, CA, USA; 2 Chao Family Comprehensive Cancer Center, University of California at Irvine, School of Medicine, Irvine, CA, USA; 3 Department of Pathology, University of California at Irvine, School of Medicine, Irvine, CA, USA; 4 Department of Molecular Biology & Biochemistry, School of Biological Sciences, University of California at Irvine, School of Medicine, Irvine, CA, USA

## Abstract

**Introduction:**

Sinus histiocytosis with massive lymphadenopathy, also known as Rosai-Dorfman Disease, is a rare and benign source of lymphadenopathy first described in 1969, which mimics neoplastic processes. This disease commonly presents in children and young adults with supra-diaphragmatic lymphadenopathy or extranodal lesions consisting of tissue infiltrates composed of a polyclonal population of histiocytes. Since its description greater than 400 cases have been described, sometimes in patients with a variety of treated and untreated neoplastic diseases. However, the literature contains reports of only 19 cases of Rosai-Dorfman Disease in association with lymphomas, Hodgkin's or non-Hodgkin's. The majority of these cases have the two diagnoses, malignant lymphoma and Rosai-Dorfman Disease, separated in time. Interestingly, infradiaphragmatic lymphadenopathy was a feature in the majority of previously reported cases of Rosai-Dorfman Disease and non-Hodgkin's lymphoma.

**Case presentation:**

This report provides details of a case with co-existing sinus histiocytosis with massive lymphadenopathy and diffuse large B cell non-Hodgkin's lymphoma. This case is the fifth described case of simultaneous Rosai-Dorfman Disease and concurrent non-Hodgkin's lymphoma. Unfortunately, the diagnosis of a clinically aggressive diffuse large B cell lymphoma was made at autopsy. The aggressive biological behavior of the diffuse large B cell lymphoma in this patient may have been related to the underlying immune dysregulation believed to be part of the pathophysiology of Rosai-Dorfman Disease.

**Conclusion:**

Taken together this report and the preceding reports of Rosai-Dorfman Disease and non-Hodgkin's lymphoma suggests that in cases with a diagnosis of Rosai-Dorfman Disease in the setting of prominent infradiaphragmatic lymphadenopathy, clinicians should maintain a high index of suspicion for the presence of occult non-Hodgkin's lymphoma especially if the clinical course is atypical for classic Rosai-Dorfman Disease.

## Introduction

Sinus histiocytosis with massive lymphadenopathy (SHML), also known as Rosai-Dorfman Disease (RDD), is a rare entity first described in 1969 [1] that belongs to a group of non-malignant histiocytic disorders in which there is a pathologic increase in the number of histiocytes, mainly mononuclear phagocytic cells and the antigen-presenting cells of bone marrow origin [2], in nodal or extra-nodal sites. This entity frequently mimics a malignant neoplasm, however the clinical course can be variable ranging from spontaneous regression, to protracted periods of stable lymphadenopathy, to the less frequent observation of progressive lymphadenopathy [3]. Symptomatic cases generally respond to mild or limited therapy such as steroid treatment, although surgery, radiation therapy and cytotoxic therapy have also been used [3]. The etiology is still unknown although genetic, infectious, and inflammatory etiologies have been postulated. This report details a case of Rosai-Dorfman disease occurring concurrently with diffuse large B cell non-Hodgkin's lymphoma and reviews the literature with respect to sinus histiocytosis with massive lymphadenopathy/Rosai-Dorfman disease particularly with respect to the previously reported cases of RDD with non-Hodgkin's lymphoma and the association with infra-diaphragmatic or retroperitoneal lymphadenopathy.

## Case presentation

A 33 year-old Hispanic female patient with no significant past medical history was seen in the outpatient clinic. She presented with a three month history of increasing abdominal discomfort, resulting in a sense of fullness, decrease oral intake, lethargy and extensive weight loss of 15 Kilograms, a two month history of a dry nonproductive cough, which was worse in the supine position, low grade fevers without rigors, and mild night sweats that did not reach the criteria for classic night sweats. At presentation she was afebrile, mildly tachycardic (98–109 bpm), normotensive and not distressed. Abdominal examination revealed midline fullness slightly more prominent on the left than the right side with some tenderness to palpation in all four quadrants. Peripheral lymphadenopathy was not appreciated by palpation and the remainder of the physical examination was unremarkable. Laboratory data revealed a normochromic microcytic anemia (hemoglobin at 10.5 g/dl) and hypoalbuminemia (1.4 mg/dl) with a normal liver enzyme panel. Electrolytes, white blood cell count with differential & smear and coagulation laboratories were all within institutional limits.

**Figure 1:**
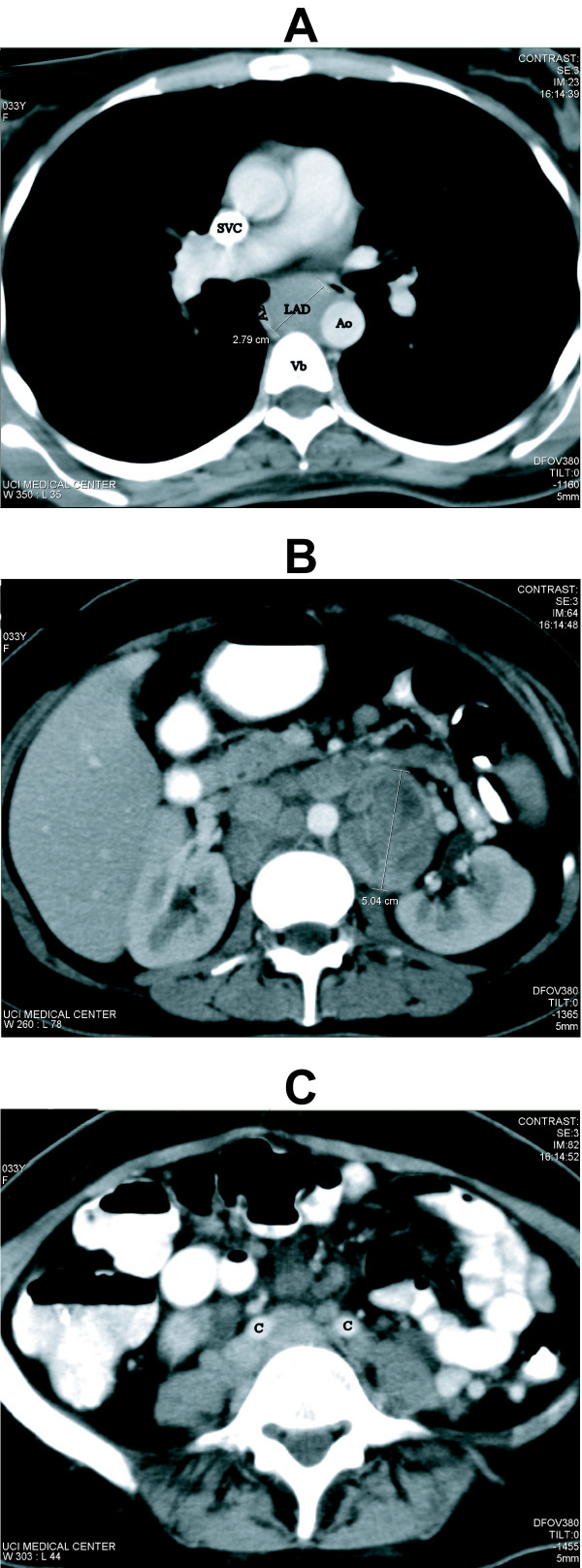
**Computed Tomography at Presentation**. **Panel A**. Chest CT demonstrating enlarged lymph node (LAD) in the azygoesophageal recess measuring 2.8 cm × 2.3 cm. Note the anterior deviation of the trachea. Az (azygous vein), Ao (aorta), Vb (vertebral body), SVC (superior vena cava). **Panel B**. Abdominal CT demonstrating multiple enlarged centrally necrotic retroperitoneal lymph nodes. The largest conglomerate, left periaortic lymph nodes, measured 5.5 cm × 5.0 cm. White arrows point to additional masses with central necrosis. **Panel C**, Abdominal CT demonstrating bilateral common iliac lymphadenopathy (C = common iliac artery).

Imaging studies revealed the development of extensive, primarily abdominal lymphadenopathy. An abdominal ultrasound revealed extensive adenopathy in the periaortic region, with a prominent hypoechoic mass at the left renal pelvis and mild splenomegaly. A report of an abdominal ultrasound performed five months earlier at a separate facility was reported as being negative for pathologic findings. A CT scan of the chest, abdomen and pelvis was performed revealed massive central lymphadenopathy with no peripherally located lymphadenopathy. A large lymphoid mass, 2.8 × 2.3 cm, was identified in the azygoesophageal recess, Figure 1 Panel A, along with limited aortopulmonary and pretracheal lymph nodes all measuring less than 1 cm in maximal dimension. In the abdomen, there were multiple enlarged centrally necrotic retroperitoneal lymph nodes, the largest conglomerate was in the left periaortic area measuring 5.5 cm in maximal dimension, Figure 1 Panel B, and there was evidence of significant bilateral common iliac lymphadenopathy, Figure 1 Panel C. There was no evidence of pelvic sidewall or inguinal lymphadenopathy. Also the patient was noted to have mild hepatomegaly, splenomegaly of 13.4 cm, mild right hydronephrosis and bilateral hydroureter.

The patient tested negative, by serology and culture, for other causes of massive lymphadenopathy, which included *Coccidiomycosis*, *Aspergillus*, *Histoplasmosis*, *Blastomyces, Cryptococcu*s, *Legionella *and *Tuberculosis*. Serologic markers for EBV (both IgG & IgM) and CMV were positive, but did not support an acute infection by either agent. Serologic screens for HIV and HSV type I, were negative. Although the patient had elevated levels of C-reactive protein (>20+ mg/L) and serologic reactivity for anti-smooth muscle antibodies, there were no anti-mitochondrial, anti-nuclear, or anti-neutrophil cytoplasmic (both C & P) antibodies detected. Serum AFP, CA-125 and CA 19-9 levels were within normal limits.

**Figure 2:**
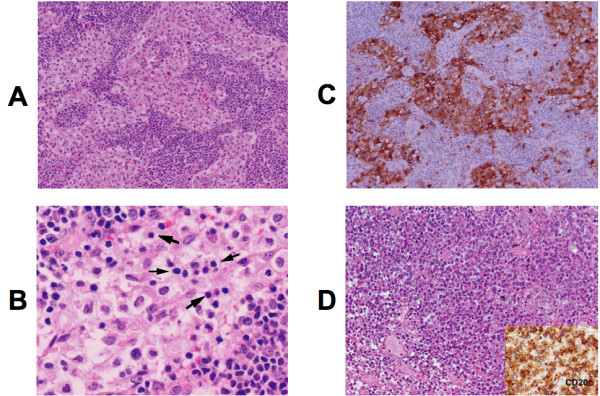
**Photomicrographs of excisional lymph node biopsy**. **Panel A**, Lymph tissue demonstrating expanded sinuses with histiocyte infiltration and distorted architecture. (HE stain, Original magnification 40 ×). **Panel B**. A higher magnification view demonstrating lymphocytes inside histiocytes (HE stain, Original magnification 400 ×). Notice pale cytoplasm, ovoid nuclei with a single nucleolus and open chramatin of histiocytes in contrast to lymphocytes with smaller round nuclei and dense chromatin. Examples of emperipolesis are noted by arrows; grey arrows identify examples of single lymphocytes within histiocytes and a curvilinear group of five lymphocytes are bracketed by the black arrows. **Panel C**. This panel depicts immunohistochemical evaluation for S-100. The large histiocytes are immunoreactive to S100 with both cytoplasmic and nuclear staining and the unstained cells in the cytoplasm of histiocytes are engulfed lymphocytes (Immunohistochemical stain, Original magnification 40×). **Panel D**. The panel contains a representative HE stained section from tissue obtained from the abdominal mass at autopsy depicting the diffuse large B cell non-Hodgkin's lymphoma diagnosed, the insert depicts positive anti-CD20 immunohistochemical staining, (Original magnification 100 ×).

The patient underwent an excisional biopsy of inferior retroperitoneal lymph nodes by a laparoscopic procedure. Three lymph nodes measuring 1.2 × 0.9 × 0.5 cm, 1 × 0.8 × 0.8 cm, and 0.9 × 0.9 × 0.7, respectively, were obtained from this procedure. Flow cytometry was performed on dispersed cells from the tissue sample and did not demonstrate a monoclonal population of lymphocytes. Histologic sections of the lymphatic tissue indicated increased prominence of the sinuses with marked histiocytic proliferation resulting in effacement of architecture containing large histiocytes with vacuolated cytoplasm, Figure 2 Panel A. Some histiocytes contained lymphocytes in their cytoplasm (emperipolesis), examples of which are highlighted by arrows, Figure 2 Panel B. The Immunohistochemical staining revealed positive S-100 staining of histiocytes, Figure 2 Panel C, and absent CD1a, CD15, CD30 staining; features consistent with RDD. No evidence of atypical or monoclonal lymphocytic proliferation was found.

The patient's hospital course followed an inexorable decline with markedly decreased PO intake secondary to abdominal discomfort, persistent night sweating, fevers and tachycardia. The patient required multiple erythrocyte transfusions with no evidence of hemolysis, overt hemorrhage, or thrombocytopenia. A bone marrow biopsy revealed a slightly hypercellular marrow with no diagnostic abnormalities and cultures that remained negative. Post, excisional biopsy, the patient was cared for in the intensive care unit (ICU) where parenteral nutrition was started and respiratory support was provided, ultimately requiring intubation and ventilator support. Treatment with decadron at 40 mg IV daily was initiated after pathologic evaluation of the excisional biopsy. This treatment provided transient symptomatic improvement, but the patient experienced increasing abdominal discomfort, development of icteris (total bilirubin = 20.6, direct bilirubin = 12, with an albumin of 1), and increasing pulmonary infiltrates within a week. An extensive infectious disease work-up including multiple cultures along with bronchial alveolar levage (BAL) fluid cultures revealed pathogenic organisms after more than a week in the ICU. The identified organisms included, oral candidiasis, actinomycosis in blood, and on repeat BAL both 1+ HSV and 1+ EBV. Despite broad-spectrum antibiotics, the patient progressed to acute renal insufficiency, anasarca, and refractory hypotension leading to death over several days.

At autopsy, the retroperitoneal & mediastinal lymphadenopathy had increased in size drammatically, encased vascular structures and was contiguous with the pancreas displacing the duodenum ventrally. Histologic examination showed extensive necrosis and regions of CD20 & CD79a positive staining cells consistent with diffuse large B cell lymphoma (DLBCL), Figure 2 Panel D. Markers for T lymphocytes & histiocytes were negative within this mass. The DLBCL also involved sections of thyroid. Pulmonary actinomycosis or disseminated infectious processes were not demonstrated. Thus, the patient was considered to have both DCBCL and RDD, although the former predominated at autopsy.

## Discussion

Sinus histiocytosis with massive lymphadenopathy (SHML) or Rosai-Dorfman Disease (RDD) was first described in 1969 [1], since then >400 cases have been reported in the RDD registry [2,3]. This disease usually affects individuals in childhood and early adulthood but has a predilection for males of Caucasian and African descent in their twenties [2,3]. It is generally considered a benign histiocytic disorder. Other disorders in this group include dendritic cell-related histiocytoses (like Langerhans cell histiocytosis), macrophage-related histiocytoses (like hemophagocytic lymphohistiocytosis), and histiocytosis from underlying malignancy or infection. However, this entity has a unique histopathologic appearance that distinguishes it from other processes in this group. RDD is diagnosed on histologic or cytologic basis, when evidence of abnormal amounts of histiocytes infiltrating lymph tissue is observed [2,3]. The lymphoid tissue has massively engorged sinuses and/or interfollicular areas with infiltrating cells, which stain positive for S-100 protein [4,5]. Immunohistochemical staining studies clearly identify RDD histiocytes as a separate process from T or B lymphoid population [4]. These cells are described as having abundant pale cytoplasm, round-to-oval nuclei with a single nucleolus and with evidence of lymphocytophagocytosis or emperipolesis. The etiology of RDD is still unknown. It has been noted to occur sporadically, with occasional clustering, which suggests a genetic or an infectious component [2]. It has been suggested that this disease is linked to a reactive disorder since it arises from circulating mononuclear cells and there is an increase in auto-immune antibodies in some affected individuals [2,3]. Epstein-Bar virus and human herpes virus 6 has been purposed as the infectious agent and recently other diseases has been implicated like varicella zoster virus, cytomegalovirus, *Brucella*, and *Klebsiella *[2]. It has also been postulated that the development of RDD may be driven by disregulated cytokine expression [2].

RDD commonly presents as bilateral painless cervical and paratracheal lymphadenopathy, which can be massive, in 90 per cent of the cases [2]. Other less common sites of involvement are inguinal, axillary, mediastinal and upper paraaortic lymph nodes. Common sites of extranodal involvement include skin, upper respiratory tract, Waldeyer's ring, and bone [2,3]. Rarely does this disease cause systemic symptoms or organ dysfunction; the bone marrow, liver and spleen are usually spared. There are nonspecific symptoms that may herald the onset of RDD such as fevers and pharyngitis, which can be accompanied by pain, tenderness, malaise, night sweats, weight loss and anemia [2,3,5]. The course of this disease can remain indolent for years and can spontaneously regress without treatment and generally does not relapse. A few reported cases became progressive and required treatment secondary to life threatening complications or organ dysfunction; prognosis is poor if there is involvement of kidneys, lungs or wide spread nodal dissemination [5,6]. There is no agreed upon treatment protocol. In most patients it is acceptable to use a watch and wait approach. Progressive or symptomatic disease is initially treated with a high dose steroids followed by surgery, radiation and/or single agent cytotoxic chemotherapy in refractory cases [5] with the latter having relatively poor response rates. It is intriguing that this patient had a transient improvement with the initiation of steroid therapy, possibly due to the effect on her RDD and or the non-Hodgkin's lymphoma, however this was short lived suggesting that the underlying debilitation, immune compromise from both diagnoses, and the aggressive growth characteristics of what proved to be an unrecognized diffuse large B cell lymphoma led to her demise.

This case report demonstrates a rare presentation of Rosai-Dorfman disease (RDD) with Large B-Cell lymphoma. An extensive search of the literature reveals only 19 reported cases of lymphoma (Hodgkins and non-Hodgkins) occurring in patients also diagnosed with RDD [7-16], Table 1. There is temporal dissociation of these two diagnoses in the majority of these cases with only 8 having concordant presentation of both RDD and lymphoma although NHL was diagnosed at the same time as RDD in four cases [7-10] with most of the cases reporting that RDD and NHL presented in separate nodal locations [9]. Interestingly, prominant retroperitoneal lymphadenopathy has been a frequent observation in these cases, as was seen in this patient, rather than the typical supra-diaphragmatic lymphadenopathy [2] typically seen with RDD. Together, this suggests that a diagnosis of RDD in the presence of massive retroperitoneal lymphadenopathy should be cause for maintaining a high clinical index of suspicion for NHL and for consideration of additional biopsy of separate nodal areas.

**Table 1:** 

#		Lymphoma histologic diagnosis	Lymphoma site(s)	SHML/RDD site(s)	Time Interval between Dx of SHML and initial lymphoma Dx	Additional Comments
1	Lu, et al (9)	Follicular Center NHL	Diffuse adenopathy & bone marrow	Left Inguinal lymph node	Concurrent	
2		Diffuse mixed small and large cell NHL	Extensive Mesenteric lymph nodes	Left inguinal lymph node	SHML diagnosed 16 yr later	16 yr later concurrent follicular center NHL left inguinal lymph node
3		Lymphocyte predominant Hodgkins Disease	Left Axillary lymph nodes	Left Axillary lymph nodes	Concurrent with HD see additional comment	14 yr later diffuse large B cell NHL – retroperitoneal lymph nodes
4	Shoda, et al (10)	Diffuse large B cell NHL	Bone marrow	Cervical lymph nodes	Concurrent	
5	Garel, et al (7)	Anaplastic Large Cell NHL	Retroperitoneal, pelvic lymph nodes	Mediastinal Lymph nodes	Concurrent	
6	Focar, et al (3)	Immunoblastic NHL CNS	Central Nervous System	cervical and axillary lymph nodes	SHLM preceded NHL by 8 mo	
7		NHL (NOS)	Unknown	Unknown	Unknown	
8		NHL (NOS)	Unknown	Unknown	Unknown	
9	Rangwala, et al (11)	Small non-cleaved (non-Burkitt's) lymphoma	kidney & palate	Inguinal lymph nodes	SHLM preceded NHL by >4 yr	
10	Krzemieniecki, et al (13)	High grade NHL (NOS)	axillary lymph nodes	cervical lymph nodes	SHLM preceded NHL by 5 yr	
11	Menzel, et al (14)	High grade NHL (NOS)	cervical lymph nodes	Axillary & inguinal lymph nodes and SC tissues	SHML diagnosed 6 yr later	
12	Kodura, et al (8)	T cell NHL	cervical lymph nodes	Retroperitoneal lymph nodes	SHLM preceded NHL by 10 yr	

## Conclusion

Simultaneous RDD and lymphoma remains a rare entity. However, the diagnosis of RDD in the presence of prominent subdiaphragmatic lymphadenopathy should raise the index of suspicion for concomitant lymphoma especially in the setting of limited response to steroid therapy.

## Abbreviations

AFP: Alpha Fetal Protein; BAL: Bronchial Alveolar Lavage; bpm: beats per minute; CD: Cluster determinant; cm: centimeter; CMV: cytomegalovirus; CT: Computed Tomography; dl: deciliter; DCBCL: Diffuse Large B Cell Lymphoma; EBV: Epstein Barr Virus; g: gram; HD: Hodgkin's disease; HIV: Human Immunodeficiency Virus; HSV: Herpes Simplex Virus; ICU: Intensive Care Unit; Ig: immunoglobulin; IV: intravenous; L: liter; mg: milligram; mm: millimeter; mo: month; NHL: non-Hodgkin's Lymphoma; NOS: not otherwise specified; PO: per oral; SHML: Sinus Histiocytosis with Massive Lymphadenopathy; RDD: Rosai-Dorfman Disease; yr: year(s).

## Competing interests

The authors declare that they have no competing interests.

## Authors' contributions

JM participated in the care of this patient and preparation of the manuscript. XZ performed the pathologic examinations and provided the photomicrographs. EN supervised the care of this patient and the preparation of the manuscript.

## Consent

Written informed consent was obtained from the next of kin for publication of this case report and accompanying images. A copy of the written consent is available for review by the Editor-in-Chief of this journal.
